# Biomaterials Based on Bacteria for Cancer Clinical Therapy

**DOI:** 10.34133/bmr.0350

**Published:** 2026-04-13

**Authors:** Chongyu Liang, Xiaoming Sun, Lingyu Xin, Meiting Yi, Kai Ma, Zicun Li, Hao Ran, Wei Zhu, Zenghao Wang, Jiandong Zhang

**Affiliations:** ^1^ Shandong Traditional Chinese Medicine University, Department of Radiation Oncology, The First Affiliated Hospital of Shandong First Medical University, Jinan 250014, People’s Republic of China.; ^2^ Department of Thoracic Surgery, Jinan Central Hospital, Jinan 250013, People’s Republic of China.; ^3^ Jinan Third People’s Hospital, Jinan 250132, People’s Republic of China.; ^4^ School of Preventive Medicine Sciences, Shandong First Medical University, Jinan 250117, People’s Republic of China.

## Abstract

Cancer remains a major global health burden, necessitating innovative therapies. Bacterial therapy has reemerged as a transformative strategy, particularly for the potential to overcome resistance to conventional treatments. Bacteria could selectively colonize the hypoxic, acidic, and immunosuppressive tumor microenvironment. Besides, synthetic biology has enabled these bacteria to be engineered into precise living therapeutics, capable of localized drug delivery, targeted immune modulation, and remodeling of the tumor microenvironment. This review systematically explores the mechanisms by which engineered bacteria exert antitumor effects, including direct oncolysis, immunogenic cell death, and reversal of immunosuppression. Furthermore, the synergistic potential of bacterial therapy with standard therapies is discussed. Bacteria can sensitize tumors to chemotherapy through prodrug activation or reverse microbiota-mediated chemoresistance. In radiotherapy, bacteria could act as radiosensitizers and radioprotectors while amplifying abscopal effects. Combined with immunotherapy, they effectively convert immunologically “cold” tumors into “hot” ones, enhancing the application of immune checkpoint inhibitors. The role of the gut microbiota in shaping systemic antitumor immunity is also discussed. Finally, the article critically assesses the clinical translation landscape, examining representative strains in development, addressing safety and manufacturing challenges, and highlighting future directions. This review aims to provide a foundational perspective for developing safer and more effective bacterially mediated anticancer strategies.

## Introduction

Cancer is one of the leading causes of morbidity and mortality globally [1]. Its highly complex biological characteristics impose a persistent and heavy burden on global public health and economic development [2]. Despite important advancements in conventional cancer interventions such as surgery, chemotherapy, radiotherapy (RT), and targeted therapy, numerous challenges persist in achieving long-term remission or complete cure of malignant tumors [3]. These challenges include tumor heterogeneity, drug resistance mechanisms, metastasis and recurrence, as well as dose-limiting systemic toxicity. Therefore, the urgent need for innovative therapeutic strategies that can enhance treatment efficacy while mitigating adverse effects is particularly evident [4].

**Fig. 1.**
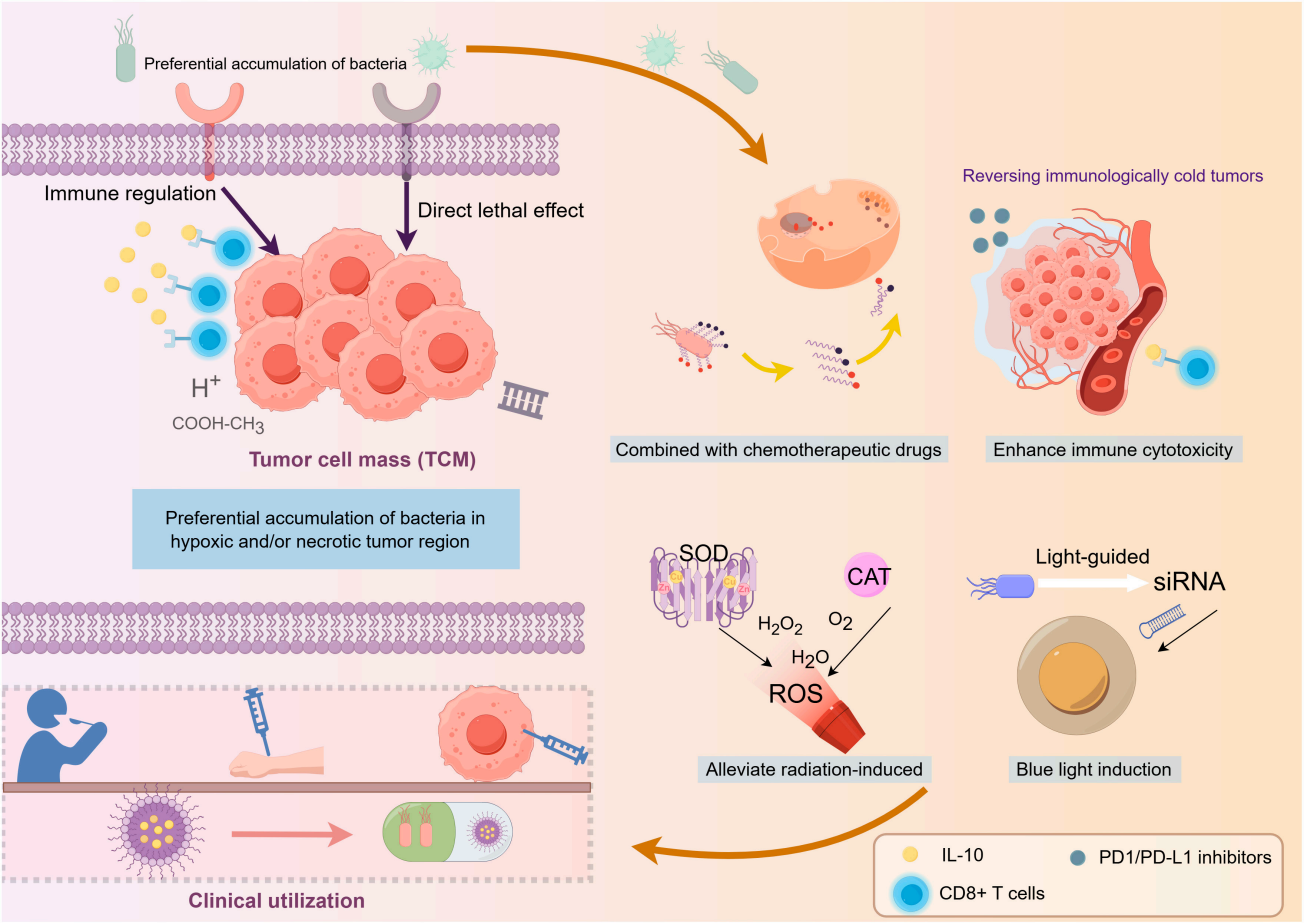
Schematic diagram of bacteria combined with multiple modalities for tumor treatment, including chemotherapy, immunotherapy, targeted therapy, radiotherapy, and photothermal therapy.

Bacteria-based tumor therapy strategies have garnered important attention due to their unique biological properties [5], with a history tracing back to the late 19th century. At that time, William Coley observed that bacterial infections could induce spontaneous tumor regression in patients, thereby pioneering the field of bacterial anticancer therapy [6]. A core advantage of bacterial therapy lies in its superior tumor-targeting capabilities: Bacteria can selectively colonize and proliferate within the tumor microenvironment (TME) [7]. As depicted in Fig. 1, solid tumors typically exhibit abnormal characteristics such as hypoxia, acidosis, nutrient deprivation, and immunosuppression [8]. While these conditions restrict the effectiveness of conventional therapies, they simultaneously provide an ideal habitat for anaerobic or facultative anaerobic bacteria. Bacteria achieve enrichment in tumor regions through chemotaxis (e.g., sensing TME metabolites like lactate) and anaerobic metabolic mechanisms, leading to local proliferation [9]. This enables drugs to reach high concentrations within the tumor while significantly reducing off-target toxicity associated with systemic exposure. This self-directed, self-replicating “living therapeutic” characteristic endows bacterial vectors with unparalleled potential for tumor targeting, a feat difficult for synthetic systems to match [9].

The rapid advancement of synthetic biology has provided powerful tools for bacteria-based tumor therapy, driving its evolution toward precision, high efficiency, and multifunctionality [10–12]. Through genetic engineering, bacteria can be engineered to synthesize chemotherapeutic agents in situ within tumors or to express specific enzymes (e.g., cytosine deaminase) that convert prodrugs (e.g., 5-fluorocytosine) into active toxins (e.g., 5-fluorouracil [5-FU]) [13]. This approach enhances the therapeutic index and mitigates systemic toxicity. Furthermore, the combination of engineered bacteria with immune checkpoint inhibitors (ICIs) (e.g., programmed cell death protein 1 [PD-1]/programmed death-ligand 1 [PD-L1] blockers) can effectively reverse the immunosuppressive TME and enhance T-cell infiltration and antigen presentation, thereby improving immunotherapy efficacy and potentially overcoming immune resistance [14,15]. Bacteria can also be designed to scavenge radiation-induced reactive oxygen species (ROS) (e.g., H_2_O_2_), protecting normal tissues from damage while simultaneously enhancing tumor radiosensitivity [16]. Moreover, synthetic biology techniques enable bacteria to achieve spatiotemporally controlled drug release. For instance, they can precisely deliver therapeutic molecules like small interfering RNA under specific stimuli, thereby opening new avenues for personalized cancer treatment [17].

In summary, this review systematically explores the multifaceted potential of bacterial therapy in cancer treatment. It particularly elucidates the advantages of localized antitumor effects and toxicity control achieved through bacteria’s inherent tumor tropism and modifications via synthetic biology techniques. The article details the latest advancements in bacteria-assisted cancer therapy, specifically highlighting the revolutionary role of synthetic biology in advancing this field and the synergistic potential demonstrated when bacterial therapies are combined with conventional treatment modalities. Furthermore, this paper also focuses on the pivotal role of the gut microbiota in modulating systemic antitumor immunity and influencing immunotherapy responses. The aim is to provide a theoretical basis and to critically evaluate the clinical translation landscape, identifying key hurdles and promising avenues for developing safer and more effective bacterially mediated strategies for cancer patients.

## Biological Principles and Engineering Strategies of Bacterial Therapy

**Fig. 2.**
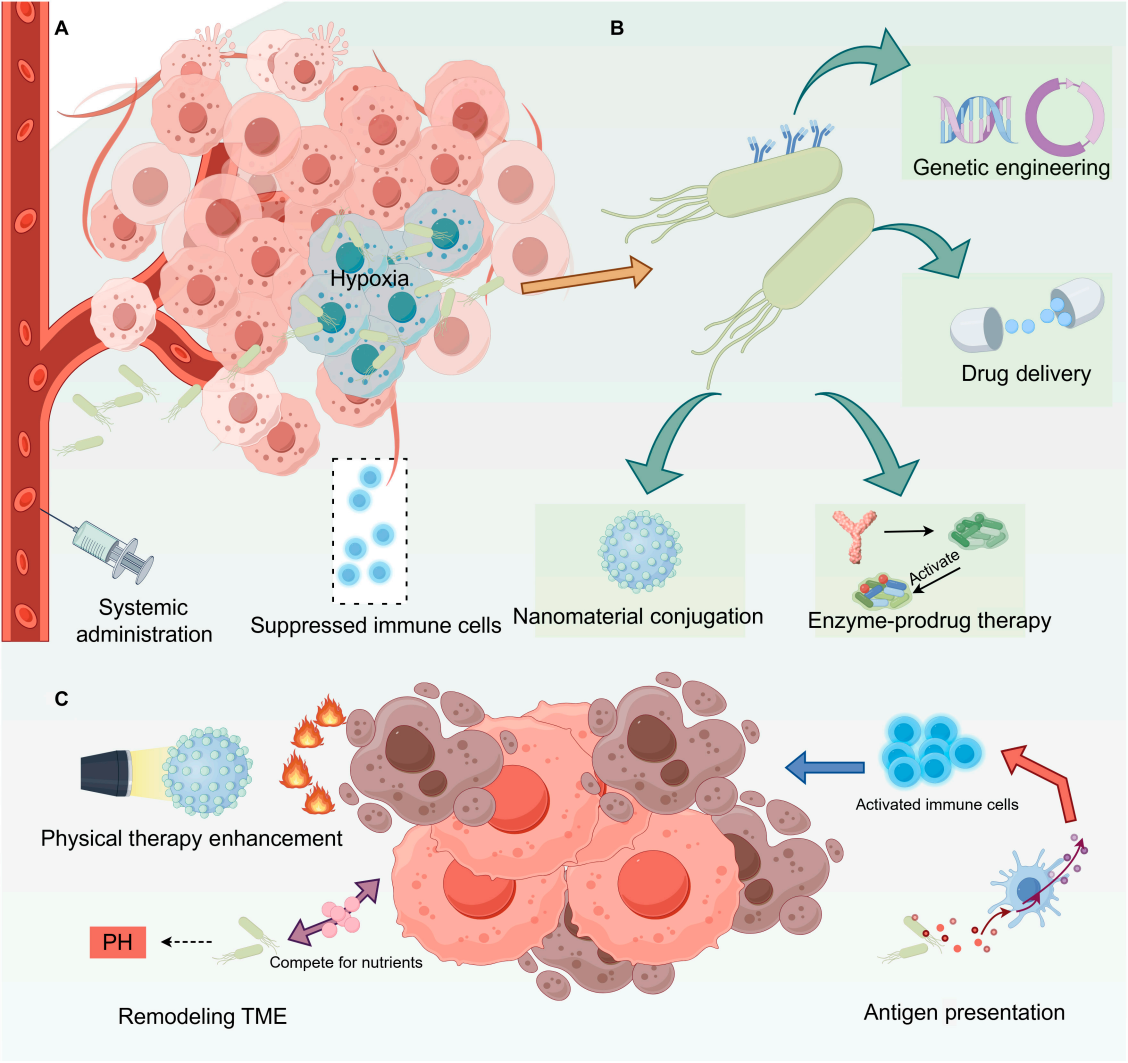
Engineered bacteria for multimodal tumor therapy. (A) Due to the enhanced permeability and retention (EPR) effect and the hypoxic, immunosuppressive tumor microenvironment, bacteria selectively colonize tumors. (B) Bacteria are engineered into living therapeutic carriers through genetic modification (e.g., secretion of therapeutic proteins), drug loading, or conjugation with nanomaterials. (C) Engineered bacteria exert antitumor effects through direct cytotoxicity, activation of innate and adaptive immunity (e.g., activation of T cells via antigen-presenting cells [APCs]), and synergistic physical therapies (e.g., photothermal therapy [PTT]).

To systematically elucidate the foundational principles, engineering strategies, and therapeutic mechanisms of bacterial therapy, we have developed a comprehensive schematic presented in Fig. 2. This figure illustrates the entire process, from the initial tumor targeting and colonization of bacteria (Fig. 2A), through the various bioengineering modifications to functionalize them as therapeutic agents (Fig. 2B), to the ultimate multimodal antitumor effects they exert within the TME (Fig. 2C).

### Bacterial biological behavior

In the TME, bacteria exhibit selective tropism and efficient colonization by sensing and responding to specific physicochemical cues [18]. The inherent complexity of the TME provides diverse environmental signals crucial for precise bacterial targeting. For instance, the severe hypoxic conditions within the tumor, tumor cell-specific metabolites (e.g., lactate and succinate), and the acidic microenvironment (low pH) collectively serve as critical chemotactic signals for bacteria [19–21]. While tumor hypoxia activates the hypoxia-inducible factor 1-alpha (HIF-1α) pathway in tumor cells [22], regulating a cascade of gene expression, bacteria directly sense the low-oxygen environment itself and its resulting metabolic alterations. Furthermore, molecules released from necrotic tumor regions (e.g., DNA and adenosine triphosphate) are also recognized by bacteria, further promoting their specific colonization in these areas [23].

Different bacterial strains possess specialized chemoreceptors and signaling pathways pivotal for tumor targeting [24]. For instance, obligate anaerobes such as *Clostridium* spp. employ specific chemoreceptors to detect low-oxygen conditions and anaerobic metabolites [25,26]. This capability directs their efficient colonization specifically to the hypoxic and necrotic regions of tumors. In contrast, *Salmonella* spp. utilize sophisticated signal transduction systems to perceive and adapt to diverse signals within the TME, such as fluctuations in pH, nutrient gradients, and redox potential [5,27,28]. This sophisticated sensing facilitates their precise targeting of tumor tissues. The high specificity of these bacterial chemoreceptors and associated signaling pathways ensures their accurate accumulation within tumor tissues [29], thereby improving the targeting precision and efficacy of subsequent therapeutic interventions. The penetration and dissemination of bacteria within tumors are influenced by multiple physicochemical factors [30]. Active motility represents a primary mechanism of bacterial migration within tumor tissue [31]; many bacteria utilize flagellar propulsion for directional movement, allowing them to actively penetrate deep into the tumor interior, particularly into oxygen-deprived and necrotic regions favorable for their growth [32]. Passive diffusion also contributes to the local spread of bacteria within the tumor [33]. However, the efficient proliferation of bacteria within tumors is predominantly contingent upon their ability to adapt to and exploit the unique physiological and biochemical conditions of the tumor [7]. This is especially true for the abundant nutrients and suitable growth environment provided by hypoxic and necrotic areas. The low-oxygen conditions and rich organic matter in these specific regions offer an ideal ecological niche for rapid bacterial growth and replication [34].

The formation of biofilms or microcolonies by bacterial populations within the tumor is a crucial mechanism for their survival and functional persistence within the TME [20,35]. Biofilms are complex microbial communities formed by bacteria secreting extracellular polymeric substances and adhering to surfaces, which provides a stable structure for bacterial colonization within tumor tissues and significantly enhances their resistance to various stressors [36,37]. Microcolonies refer to smaller, localized bacterial clusters formed within the tumor tissue [38]. The distribution of different bacterial strains within solid tumors exhibits substantial heterogeneity, intrinsically linked to the TME’s complexity [39]. The intrinsic tumor-targeting capability of certain bacterial strains is the cornerstone of their therapeutic potential, a process visually detailed in Fig. 2A. Upon systemic administration, bacteria leverage the chaotic and poorly formed tumor vasculature. This results in their passive accumulation within the tumor stroma via the enhanced permeability and retention effect. Crucially, as depicted in the figure, bacteria then actively migrate and proliferate within the deep-seated hypoxic and necrotic regions of the tumor. This unique niche not only provides an ideal anaerobic environment for their survival but also serves as a sanctuary from the host’s immune surveillance, which is often compromised in the immunosuppressive TME [40]. Furthermore, noninvasive imaging techniques to monitor bacterial distribution in patients will be crucial for dose optimization and predicting response [41].

### Classification and engineered design of therapeutic bacteria

Bacteria-mediated therapy has emerged as a promising modality in the field of cancer treatment [42]. This strategy leverages the innate biological properties of various bacterial strains, augmented by synthetic biology techniques, to achieve efficient and specific tumor-targeting therapy. Multiple bacterial strains are under extensive investigation due to their unique adaptability to the TME and intrinsic antitumor capabilities [43,44]. Building upon their natural tumor-tropic properties, synthetic biology offers a powerful toolkit to engineer bacteria into sophisticated “living drugs” capable of performing complex therapeutic tasks. The primary strategies for this functionalization are summarized in Fig. 2B.

Obligate anaerobes, such as *Clostridium* spp., efficiently colonize the hypoxic and necrotic cores of tumors—regions often refractory to conventional therapies—and exhibit intrinsic oncolytic activity [26]. Attenuated *Salmonella* spp., such as the clinically tested strain *VNP20009*, are widely studied for their pronounced tumor tropism. However, the early-phase trial of *VNP20009* monotherapy revealed limited efficacy and dose-limiting toxicities, underscoring the need for further attenuation and combination regimens. Current clinical efforts focus on using engineered *Salmonella* as vectors for localized immunomodulator or drug delivery to enhance their therapeutic index [45]. Their genetic tractability also makes them attractive vectors for multifunctional therapeutics. *Escherichia coli* (*E. coli*), a model organism with a well-established genetic system, serves as an optimal chassis for constructing complex genetic circuits and delivering therapeutic agents [46]. *Bifidobacterium* spp., known as probiotics, demonstrate favorable safety profiles, thrive in hypoxic conditions, and are amenable to oral administration, facilitating their clinical translation [47]. Other promising strains include *Listeria monocytogenes*, valued for its immunogenicity and ability to elicit antitumor T-cell responses, and *Lactobacillus* spp. [48,49], probiotics that modulate gut microbiota and enhance host immunity, positioning them as potential adjuvant therapeutics.

Synthetic biology and genetic engineering are pivotal for enhancing the safety, efficacy, and precision of bacterial therapeutics [5,7,50]. Key strategies encompass several critical aspects: biocontainment and safety, tumor targeting, controlled gene expression and therapeutic delivery, and the development of integrated multifunctional platforms. First, biocontainment and safety are enhanced through virulence attenuation via genetic deletions, such as *aroA* knockout, which induces auxotrophy to limit bacterial proliferation in vivo [51], coupled with the incorporation of inducible suicide systems for precise clearance posttreatment, thereby minimizing toxicity. Second, tumor targeting is improved by engineering bacteria to modify surface receptors, express specific adhesins, or redesign chemotaxis systems, thereby strengthening their recognition of and migration toward tumor-specific signals while reducing off-target colonization [52]. Third, controlled gene expression and therapeutic delivery utilize TME-responsive promoters—activated by hypoxia, acidity, or nutrient starvation—to ensure tumor-specific, high-level production of therapeutic agents [53]. This approach is complemented by controlled-release systems, such as light- or drug-inducible genetic switches, for on-demand production of therapeutic molecules, alongside the engineering of secretory pathways to efficiently deliver cytotoxic proteins, immunomodulatory factors, and prodrug-converting enzymes [54]. Finally, integrated multifunctional platforms incorporate biosensors with feedback loops, enabling bacteria to dynamically perceive complex TME signals and adapt their therapeutic output accordingly [55]. Furthermore, the engineering of theranostic bacteria, which combine imaging functions via fluorescent or magnetic proteins with therapeutic capabilities, offers a comprehensive platform for precise cancer diagnosis and treatment. Collectively, these synthetic biology-driven strategies substantially advance bacteria-mediated therapies by systematically improving their safety, targeting, efficacy, and adaptability, thereby expanding their potential as a transformative modality in oncology.

### Mechanisms of tumor regulation by engineered bacteria

Engineered bacteria mediate antitumor effects through a multitude of mechanisms, leveraging not only the bacterial cells themselves but also a repertoire of bioactive components they secrete or release. A systematic understanding of these mechanisms and components is fundamental to the design and optimization of bacterial therapy. The therapeutic efficacy of engineered bacteria stems from a multipronged attack on the tumor, engaging direct killing, immune modulation, and microenvironmental remodeling, as mechanistically outlined in Fig. 2C.

The direct oncolytic capacity represents one of the most primitive antitumor mechanisms of bacteria. Specific bacterial toxins, such as hemolysins that compromise cell membrane integrity, and proteins like azurin that penetrate membranes to induce caspase-dependent apoptosis or cell cycle arrest, serve as direct cytotoxic agents [56]. Furthermore, bacteria can inhibit tumor cell proliferation by competing for essential nutrients, including glucose and amino acids, thereby starving the highly metabolically active cancer cells [57]. The accumulation of bacterial metabolites, such as short-chain fatty acids and lactic acid, can also alter the intracellular milieu of tumor cells, leading to metabolic stress and eventual cell death [58].

A pivotal function of engineered bacteria is the remodeling of the tumor immune microenvironment, effectively converting “immunologically cold” tumors into “hot” ones. This immunomodulation initiates with the activation of innate immunity. Bacterial components, known as pathogen-associated molecular patterns (PAMPs), are potent triggers: Unmethylated CpG motifs in bacterial DNA activate Toll-like receptor 9 (TLR9); flagellin activates TLR5; and lipopolysaccharide activates TLR4 [59,60]. The engagement of these PAMPs with host pattern recognition receptors (PRRs) on dendritic cells (DCs) and macrophages stimulates the production of proinflammatory cytokines like tumor necrosis factor-α, interleukin-1β (IL-1β), and IL-12, thereby initiating an antitumor immune response. Subsequently, bacteria enhance antigen presentation and adaptive immunity. Bacteria-induced tumor lysis releases tumor-associated antigens, while the bacteria themselves act as potent adjuvants, promoting DC maturation and the subsequent priming of tumor-specific CD4+ and CD8+ T cells [61,62]. Concurrently, engineered bacteria can reverse immunosuppression by diminishing the recruitment and function of myeloid-derived suppressor cells (MDSCs) and regulatory T cells (Tregs) [63] or through the localized secretion of immunostimulatory cytokines (e.g., interferon-γ [IFN-γ] and granulocyte-macrophage colony-stimulating factor) to counteract immune checkpoints.

The colonization and proliferation of certain facultative or obligate anaerobic bacteria within tumors can significantly impact the tumor vasculature. For instance, *Clostridium* spp. rapidly multiplying in hypoxic regions can physically compress or occlude tumor blood vessels, leading to ischemic necrosis and indirect tumor cell eradication. Conversely, some bacterial derivatives, such as outer membrane vesicles (OMVs), have been shown to induce transient vascular normalization, improving blood flow and drug perfusion to enhance the efficacy of coadministered therapeutics [64,65].

Additionally, bacteria function as biological competitors within the TME. Their rapid proliferation allows them to occupy ecological niches and efficiently consume oxygen and nutrients, thereby physically and metabolically suppressing tumor cell expansion through resource competition.

In summary, the antitumor efficacy of engineered bacteria is mediated by a synergistic interplay of key active components: the bacterial cells themselves, serving as living, targeted delivery vectors; bacterial DNA, acting as a natural immunostimulatory adjuvant via TLR9 signaling; bacterial metabolites (e.g., short-chain fatty acids and lactate) that modulate the local microenvironment and directly impact tumor and immune cells; bacterial toxins and secreted proteins (e.g., hemolysins, azurin, OmpA, and flagellin) that mediate direct cytotoxicity or activate specific immune pathways; and finally, engineered therapeutic agents (e.g., cytokines, antibodies, and enzymes), which represent the precision tools endowed by synthetic biology. A deep understanding of these innate mechanisms and components, coupled with advanced genetic engineering, is crucial for optimizing the precision, safety, and overall efficacy of bacterial therapies.

## Synergistic Strategies of Bacterial Therapy with Conventional Cancer Therapies

### Bacteria with chemotherapy

Building upon the foundational mechanisms of bacterial tumor colonization, immune modulation, and vascular disruption outlined in the “Mechanisms of tumor regulation by engineered bacteria” section, this chapter delves into the synergistic potential of bacterial therapy when integrated with established cancer treatments. The focus here is not on reiterating the innate capabilities of bacteria but on elucidating how these properties can be strategically harnessed to overcome the limitations of conventional therapies—such as systemic toxicity, immunosuppressive TME, and drug resistance—to enhance their efficacy and ultimately improve clinical outcomes. We will explore the combinatorial strategies with chemotherapy, RT, immunotherapy, and other emerging modalities, highlighting the mechanistic interplay and supporting preclinical and clinical evidence.

#### Prodrug enzyme conversion system

A highly effective therapeutic strategy employs bacterial prodrug-converting enzyme systems to address a major clinical challenge: the high systemic toxicity of conventional chemotherapy [66]. The core concept involves genetically engineering bacteria to express or enhance specific enzymes within the TME [67]. These enzymes convert nontoxic or low-toxicity prodrugs into potent cytotoxic agents. Since enzyme expression and activity are largely confined to bacterial colonization sites within the tumor, drug activation is spatially restricted. This localization enhances the tumor specificity of chemotherapy and reduces off-target toxicity caused by systemic drug distribution. Clinically, this approach is highly valuable, as it improves patient tolerability and quality of life while enabling more precise tumor eradication. A representative example uses engineered bacteria expressing cytosine deaminase to convert the prodrug 5-fluorocytosine, administered orally or intravenously, into the active chemotherapeutic agent 5-FU [68]. This results in localized antitumor efficacy and a significantly improved therapeutic index.

#### Localized drug delivery

Bacteria can serve as unique “living drug factories” or intelligent delivery vehicles, enabling localized and efficient delivery of chemotherapeutic agents [69]. Solid tumors pose major challenges for systemic drug delivery due to their irregular vasculature, elevated interstitial pressure, and dense cellular structure [70]. These characteristics hinder drug penetration and uniform distribution into the tumor core, limiting therapeutic efficacy. Bacteria, however, can overcome these barriers by leveraging their innate tumor tropism, active motility, and ability to proliferate in hypoxic regions. As a result, they effectively colonize and accumulate at high densities inside tumors. Once established, engineered bacteria can continuously produce and secrete therapeutic molecules—such as conventional small-molecule chemotherapeutics or drug-loaded nanoparticles—at high local concentrations. For example, the anaerobic probiotic *Bifidobacterium infantis* (*Bif*) has been developed into a biohybrid platform (Bif@DOX-NPs) for targeted delivery of doxorubicin (DOX)-loaded nanoparticles to breast tumor sites [47]. This approach not only enhances local DOX concentrations and antitumor efficacy but also significantly reduces the severe cardiotoxicity commonly associated with systemic DOX administration [71]. Thus, engineered bacterial delivery represents a promising strategy for achieving precise and effective intratumoral drug delivery while improving clinical safety.

#### Modulating the tumor microbiota to reverse chemoresistance

The development of chemoresistance poses a paramount challenge in cancer therapy [72]. Building on the inherent ability of bacteria to colonize tumors and modulate the local microenvironment (“Mechanisms of tumor regulation by engineered bacteria” section), a precise strategy to overcome chemoresistance involves the targeted editing of the tumor microbiota itself. Specifically, eliminating protumoral, resistance-promoting bacteria while fostering beneficial species can resensitize tumors to cytotoxic drugs. This approach directly addresses a major clinical hurdle—microbiota-mediated chemoresistance—by leveraging bacteria as microbial scalpels.

**Fig. 3.**
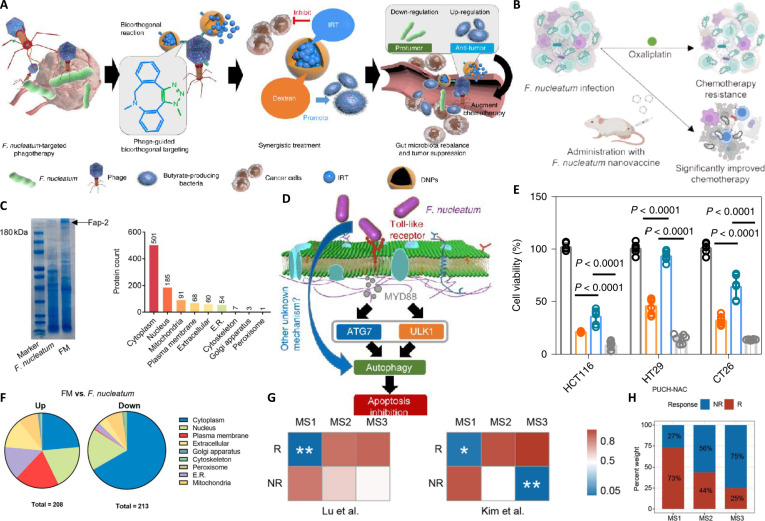
(A) Design of the phage-guided biotic-abiotic hybrid nanosystem for targeting *Fusobacterium nucleatum* (*F. nucleatum*). (B) Mechanism of bacteria-based photothermal immunotherapy. (C) Sodium dodecyl sulfate-polyacrylamide gel electrophoresis (SDS-PAGE) analysis of *F. nucleatum* whole lysate and membrane fraction (FM). (D) Schematic of the anti-*F. nucleatum* nanovaccine mechanism. (E) In vitro anticancer effect of both phage and chemotherapy (immunogenic nanodrug/nanoparticle [IDNP]). (F) Subcellular localization of Fap2 protein in *F. nucleatum* (pie chart). (G) Submap analysis of gastric cancer microbial subtypes (Msubtypes) between our cohort and responders (R)/nonresponders (NR) to immunotherapy. (H) Differences in response to neoadjuvant chemotherapy (NAC) among the Msubtypes.

A prominent clinical example is the association of *Fusobacterium nucleatum* (*F. nucleatum*) with chemoresistance. This bacterium is frequently enriched in colorectal cancer (CRC) and is an independent predictor of poor response to neoadjuvant chemotherapy (NAC), highlighting its direct clinical relevance as a therapeutic target to reverse chemoresistance [73]. A more intricate strategy involves the construction of a phage-guided biotic–abiotic hybrid nanosystem [74]. This system leverages a phage strain specifically isolated for its ability to target CRC and eliminate protumoral *F. nucleatum*. Subsequently, a bioorthogonal chemistry strategy is employed to bind immunogenic nanodrugs/nanoparticles (IDNPs)—which are designed to enhance the activity of antitumorigenic bacteria, such as butyrate-producing bacteria—to the phages preaccumulated in CRC tumors. This system thus achieves a dual therapeutic effect: First, the phage clears protumoral *F. nucleatum*, and second, the nanoparticles promote the growth of beneficial bacteria. This “suppressing the harmful and promoting the beneficial” approach to microbial remodeling synergistically enhances the efficacy of chemotherapy and reduces its side effects, offering an innovative paradigm for improving patient response and mitigating toxicity (Fig. 3A).

Research has elucidated the intricate and sophisticated mechanisms by which *F. nucleatum* induces chemoresistance [75], providing clear targets for clinical intervention (Fig. 3B). This bacterium can engage with TLRs on tumor cells via its surface proteins, such as Fap2, whose protein composition and subcellular localization are detailed in the sodium dodecyl sulfate-polyacrylamide gel electrophoresis and quantitative analysis in Fig. 3C. This interaction subsequently activates downstream signaling pathways, ultimately inducing autophagy in tumor cells. Autophagy, an adaptive cellular stress response, is frequently hijacked by tumor cells to resist chemotherapy-induced apoptosis, thereby promoting cancer cell survival and leading to clinical drug resistance (Fig. 3D). To counteract this clinically relevant *F. nucleatum*-induced resistance mechanism, researchers have developed various precise interventional strategies with promising clinical translation potential. One strategy involves developing targeted immunotherapies to eradicate the detrimental bacteria. For instance, an *F. nucleatum* nanovaccine [76] has been ingeniously designed. This vaccine comprises autologously derived *F. nucleatum* membranes combined with the potent immunostimulatory adjuvant CpG. This nanovaccine effectively activates DCs, promoting antigen presentation and thereby eliciting robust *F. nucleatum*-specific CD8+ cytotoxic T-lymphocyte (CTL) responses and humoral immunity. Such precise immune activation not only targets and eradicates intratumoral *F. nucleatum*, effectively reversing its induced chemoresistance and inhibiting tumor metastasis, but also delicately maintains the balance of the intratumor and gut microbiota [77]. This circumvents the systemic side effects and dysbiosis associated with broad-spectrum antibiotics, offering patients a safer and more precise therapeutic option (Fig. 3D).

In vitro experimental data strongly corroborate this, showing that *F. nucleatum* infection in CRC cell lines (e.g., HCT116, HT29, and CT26) significantly increases their tolerance to chemotherapeutic agents (e.g., oxaliplatin), resulting in higher cell viability and validating its capacity to induce clinical resistance (Fig. 3E) [78]. In vitro coculture experiments have also preliminarily confirmed that the combination of this phage system with chemotherapy (immunogenic nanodrug/nanoparticle) significantly reduces cancer cell viability in the presence of *F. nucleatum* [79], thereby enhancing the anticancer effect, foretelling its considerable potential for clinical application (Fig. 3E). The subcellular localization of Fap2 is further illustrated in the pie chart in Fig. 3F.

The clinical significance of these strategies is increasingly supported by accumulating patient data. Furthermore, it is noteworthy that the impact of tumor microbial subtypes (Msubtypes) on cancer therapy extends beyond chemotherapy; for instance, distinct Msubtypes in gastric cancer patients also demonstrate differential responses to immunotherapy (Fig. 3G) [80]. This further underscores the broad and profound influence of the tumor microbiome in cancer treatment, providing a crucial basis for future precision medicine and patient stratification. In CRC patients, the composition of tumor Msubtypes is significantly correlated with the response rate to NAC (Fig. 3H) [81]. This robustly confirms the immense potential of targeting and modulating the tumor microbiota to overcome chemoresistance, guide personalized therapy, and ultimately improve patient prognosis. Collectively, through these diverse molecular and immunological mechanisms, coupled with precise strategies to directly eliminate resistance-inducing microbial populations, bacteria-mediated therapies offer a novel paradigm to circumvent tumor chemoresistance. This promises to significantly broaden the applicability of chemotherapy in resistant tumors and ultimately enhance the long-term survival and quality of life for cancer patients.

### Bacteria with radiotherapy

RT is a cornerstone of oncology [82], which primarily acts by inducing DNA damage in tumor cells to inhibit their proliferation. However, the efficacy of RT is frequently limited by several factors: intrinsic or acquired radioresistance, off-target toxicity to surrounding healthy tissues, and the immunosuppressive TME, which can dampen systemic antitumor immunity. Bacteria-mediated RT has emerged as a promising strategy to overcome these challenges [83]. This approach synergistically combines the inherent tumor-targeting capability of certain bacteria with the potent cytotoxic effects of radiation, holding significant potential to enhance therapeutic outcomes. The therapeutic synergy operates through multiple mechanisms: direct radiosensitization of tumor cells, selective protection of normal tissues from radiation damage, and comprehensive remodeling of the immunosuppressive TME.

#### Radiosensitization

**Fig. 4.**
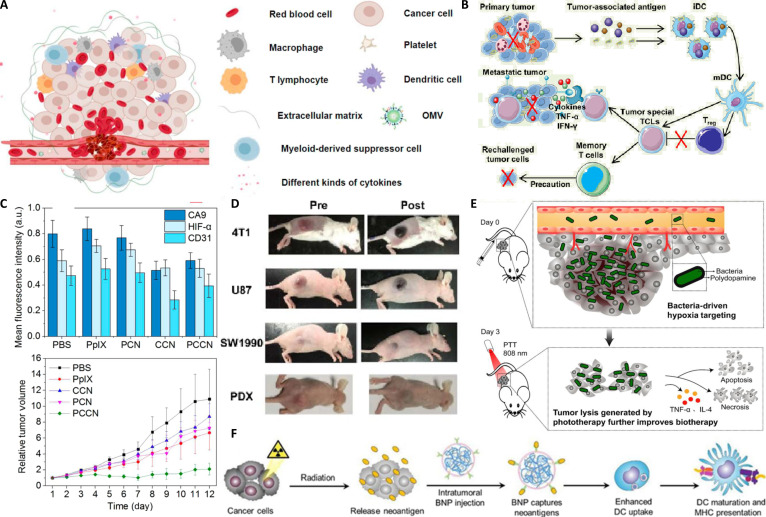
(A) Schematic illustration to show the mechanism of outer membrane vesicle (OMV)-induced extravasation of RBCs. (B) Schematic illustration of bacteria-based photothermal immunotherapy. (C) Quantitative analysis of carbonic anhydrase IX (CA9), CD31, and hypoxia-inducible factor 1-alpha (HIF-α). (D) Photographs of BALB/c mice bearing 4T1, U87MG, SW1990, and patient-derived xenografts (PDX) tumors. (E) Tumor cell lysis generated by pDA-VNP-based photothermal therapy further improves bacteria-mediated biotherapy. (F) Schematic of the in situ vaccine effect elicited by combined radiotherapy (RT) + bacteria-based nanoparticles (BNPs).

Bacteria can significantly enhance RT efficacy through both direct and indirect radiosensitizing mechanisms [84,85], to maximize tumor cell death while minimizing damage to healthy tissues. One key mechanism involves the ability of bacteria to induce or thrive within hypoxic regions, thereby sensitizing otherwise radioresistant areas of the tumor. Anaerobic or facultative anaerobic bacteria colonizing the tumor can consume oxygen and exacerbate intratumoral hypoxia. Although oxygen is typically a radiosensitizer, the selective proliferation of bacteria in severely hypoxic zones—coupled with their capacity to alter tumor metabolism—can render these regions more susceptible to radiation. Additionally, bacteria can serve as producers or carriers of radiosensitizing agents [83]. Genetically modified bacteria can be designed to secrete small molecules that directly enhance tumor cell sensitivity to radiation [86]. These molecules may inhibit DNA repair, generate ROS, or disrupt signaling pathways that promote radioresistance. For instance, bacterial metabolites or enzymes can interfere with HIF-1α or carbonic anhydrase IX [87], both of which are overexpressed in hypoxic tumors and contribute to treatment resistance. First, they modulate tumor vasculature. The dense and dysfunctional tumor vasculature impedes drug delivery and creates hypoxic regions, which are radioresistant. Specific bacterial strains or their derivatives, such as OMVs, can induce either transient vascular normalization—improving oxygen and drug perfusion—or direct vascular disruption, leading to tumor infarction [64]. For instance, certain OMVs promote red blood cell extravasation within tumors, a process that may improve tumor blood flow and local drug accumulation (Fig. 4A). Such vascular modulation critically influences intratumoral oxygen levels, a key determinant of radiosensitivity [83].

Beyond direct effects, bacteria can enhance RT by inducing immunogenic cell death (ICD) [88]. ICD converts the irradiated tumor into an in situ vaccine, stimulating a systemic antitumor immune response, including the abscopal effect. Although RT alone can trigger ICD, its combination with bacteria markedly amplifies this process. Through PAMPs and danger-associated molecular patterns released during bacterial lysis or metabolic activity, bacteria further activate antigen-presenting cells, such as DCs. This enhances antigen presentation and primes antitumor T cells. Figure 4B conceptually depicts how bacteria-mediated photothermal immunotherapy stimulates immunity and inhibits primary, metastatic, and rechallenged tumors, aligning with the systemic immune effects observed in related studies [89]. As illustrated in Fig. 4C, intratumoral injection of various materials—potentially including bacteria—can quantitatively modulate carbonic anhydrase IX and HIF-1α levels, thereby affecting radiosensitivity. To overcome these limitations, innovative combination strategies have been explored. For example, 1 study combined *VNP20009* with dopamine-mediated photothermal therapy (PTT). Upon near-infrared (NIR) laser irradiation, the pDA-VNP hybrids generated localized heat, achieving effective tumor ablation [90]. A single treatment cycle led to complete tumor eradication without recurrence or metastasis, underscoring a synergistic effect between the bacterial therapy and the photothermal agent. Furthermore, this bacteria-based PTT stimulated robust antitumor immunity. The tumor cell lysis induced by pDA-VNP PTT is proposed to further amplify bacteria-mediated biotherapy (Fig. 4E), and combining this approach with immune checkpoint blockade potentiated the immune response, effectively controlling ascites tumor growth. This immune activation is critical for systemic outcomes, particularly the abscopal effect, wherein irradiation of a primary tumor leads to regression of nonirradiated metastases—a rare but desirable clinical phenomenon [91]. Figure 4F schematically illustrates the in situ vaccine effect elicited by combining RT with bacteria-based nanoparticles (BNPs), showing how radiation-induced tumor cell death and bacterial stimulation synergize to generate a potent antitumor immune response. One representative approach involves engineered *Salmonella* encapsulated in cationic polymer nanoparticles, which are injected into tumor tissue [92]. These motile bacteria can capture antigens released after RT and transport them to the tumor periphery, activating DCs. Experimental data confirm that this strategy increases DC numbers in vitro and prolongs survival in multiple mouse tumor models, indicating enhanced antigen presentation and antitumor immunity. Ultimately, this approach counteracts TME, which often impedes effective antigen recognition and presentation following RT. Furthermore, recent advancements in nanotechnology offer complementary strategies to alleviate tumor hypoxia. For instance, oxygen-supplied nanomaterials have been developed to sustainably release oxygen within the TME, thereby potentiating not only RT but also enhancing the infiltration and function of CTLs, which is crucial for effective immunotherapy [93,94].

#### Radioprotection and toxicity mitigation

Conventional RT faces a major clinical challenge: the unavoidable irradiation of surrounding healthy tissues, which causes acute and late toxicities. These adverse effects diminish patients’ quality of life and often restrict the radiation dose that can be safely delivered to the tumor [83,95]. To address this issue, engineered bacteria have emerged as a sophisticated strategy to protect normal tissues from radiation injury while maintaining tumor radiosensitivity. These bacteria can be programmed to express antioxidant enzymes—such as superoxide dismutase and catalase—that scavenge ROS produced during irradiation in healthy tissues [96]. Importantly, through systemic targeting or localized delivery, the bacteria can be designed to specifically colonize or proliferate within normal tissues near the tumor site. This localized enzyme expression shields healthy cells from oxidative stress, thereby mitigating radiation-induced complications such as inflammation, fibrosis, and organ dysfunction. In contrast, the TME—often marked by severe hypoxia and distinct metabolic profiles—can be intentionally left unprotected or further sensitized to radiation by bacterial activity. This dual approach of radiosensitizing tumors while radioprotecting normal tissues promises to enhance the therapeutic ratio of RT, facilitating the delivery of higher and more effective tumoricidal doses with minimized systemic toxicity.

#### Remodeling the tumor microenvironment to enhance radiotherapy

Beyond direct radiosensitization and radioprotection, bacteria significantly enhance RT efficacy by comprehensively modulating TME [97]. The TME often constitutes a physical and immunological barrier that limits effective radiation delivery and response. Bacteria can strategically alter this hostile milieu through 2 primary, interconnected mechanisms: vascular modulation and immune reprogramming. First, bacteria exert a profound influence on the tumor vasculature. The dense, chaotic, and often dysfunctional vasculature within solid tumors contributes to hypoxia, which is a major cause of radioresistance. Certain bacterial strains or their derivatives, such as OMVs, can induce transient vascular normalization. This process improves blood perfusion and oxygen delivery to previously hypoxic tumor regions, thereby directly sensitizing these areas to radiation-induced damage. Improved perfusion also facilitates the delivery of systemically administered therapeutics. Conversely, the rapid proliferation of obligate anaerobes like *Clostridium* spp. within hypoxic and necrotic cores can physically compress or occlude tumor blood vessels, leading to ischemic necrosis. This vascular disruption synergizes with RT by eradicating tumor cells through a complementary mechanism. Second, and perhaps more critically, bacteria are potent remodelers of the immunosuppressive TME [98]. Although RT can induce a proinflammatory response, this effect is frequently suppressed by the immunosuppressive TME, which is rich in Tregs and MDSCs [99]. Through their persistent presence and secretion of immunomodulatory factors, bacteria can repolarize tumor-associated macrophages toward the antitumor M1 phenotype, recruit CTLs, and inhibit immunosuppressive cell populations. This immune reprogramming converts immunologically “cold” tumors, typically resistant to immunotherapy, into “inflamed” or “hot” tumors, thereby synergizing with and augmenting RT-induced antitumor immunity [100]. Through their persistent presence and the secretion of immunomodulatory factors, bacteria can repolarize tumor-associated macrophages from the protumor M2 phenotype toward the antitumor M1 phenotype. They also recruit CTLs and natural killer cells into the tumor bed. Concurrently, bacteria can inhibit the recruitment and function of immunosuppressive cell populations like Tregs and MDSCs. This comprehensive immune reprogramming creates a permissive environment where radiation-induced tumor antigens are more effectively presented, leading to the priming and activation of a robust, systemic antitumor immune response. This synergy can potentiate the abscopal effect, where irradiation of 1 tumor site leads to regression of metastases at distant, nonirradiated sites.

In summary, by reprogramming both the vascular architecture and the immune contexture of the TME, bacteria transform RT from a primarily localized cytocidal treatment into a potent in situ vaccine strategy. This multifaceted modulation addresses key limitations of RT—hypoxia and immunosuppression—thereby significantly amplifying its therapeutic efficacy. These mechanistic insights pave the way for the rational design of innovative combination therapies.

#### Preclinical and clinical advances in bacteria-mediated radiotherapy

The TME-modulating mechanisms described above provide a solid foundation for combining bacteria with RT. This synergy has been investigated across various preclinical models and is advancing into early-phase clinical trials, demonstrating both the promise and the challenges of this approach. An early study by Jiang et al. [101] demonstrated that preirradiated *E. coli*, combined with external beam RT (21 Gy), significantly reduced colon tumor volumes and suppressed lung metastasis in murine models. This work provided initial proof that bacterial components could synergize with radiation. However, translating live bacterial therapies faces challenges, as evidenced by the phase I clinical trial of the anaerobic *Salmonella typhimurium* strain *VNP20009* [18]. Despite its intrinsic tumor tropism, the trial was terminated due to insufficient antitumor efficacy and dose-limiting toxicities, highlighting the need for improved safety and potency.

In the context of aggressive malignancies like pancreatic cancer, Minden et al. [102] developed a radiolabeled *Listeria* strain by conjugating a radioactive rhenium isotope to attenuated *Listeria*. This platform leveraged *Listeria*’s tumor-targeting ability to deliver targeted internal RT. The radiolabeled *Listeria* construct selectively delivered radiation to metastatic sites, sparing primary tumors from excessive exposure and protecting normal tissues, showing great potential for preventing recurrence and metastasis with minimal systemic toxicity. A critical limitation of conventional RT is that the abundant tumor antigens released posttreatment often fail to elicit effective immune responses due to the immunosuppressive TME. To address this, Wang et al. [103] devised a strategy using *Salmonella* encapsulated in antigen-absorbing cationic polymer nanoparticles. Injected intratumorally, these bacteria leveraged their motility to capture RT-released antigens and transport them to the tumor periphery, thereby activating DCs. This approach aimed to transform “cold” tumors post-RT by relocating antigens to an immunologically active zone. Experimentally, this method increased DC activation in vitro and prolonged survival in multiple mouse tumor models in vivo, indicating enhanced systemic antitumor immunity. In addition, there is the PTT therapy mentioned above. Bacteria deliver photothermal agents to the tumor site, and under NIR laser irradiation, localized high temperature achieves effective tumor ablation. This single treatment cycle leads to complete tumor clearance in the model and stimulates a strong antitumor immune response, which is further enhanced when combined with immune checkpoint blockade. In summary, the field of bacteria-mediated RT is evolving from simple coadministration toward sophisticated bioengineering and combination strategies. While early clinical trials underscored the challenges of safety and efficacy, recent preclinical advances—employing genetically engineered bacteria, radio-labeled vectors, antigen-capturing systems, and multimodal combinations—offer promising avenues to overcome these hurdles. Future success will hinge on the rational integration of these engineered bacterial platforms with RT and other modalities like immunotherapy, guided by a deep understanding of TME biology and patient-specific factors.

### Bacteria with immunotherapy

The integration of bacteria with immunotherapy represents a paradigm shift in oncology, offering a potent strategy to overcome the limitations of standalone immunotherapeutic agents, particularly ICIs. While the “Mechanisms of tumor regulation by engineered bacteria” section has detailed the fundamental mechanisms by which engineered bacteria intrinsically modulate antitumor immunity—such as via PAMP–PRR signaling, antigen presentation, and immunosuppressive cell reprogramming—this section will focus on their synergistic potential when combined with existing immunotherapies [104]. We will elaborate on how bacteria can remodel the immunosuppressive TME to enhance ICI efficacy, amplify systemic antitumor immune responses, and explore the critical role of the gut microbiota in shaping immunotherapy outcomes.

#### Remodeling the tumor immunosuppressive microenvironment

The profound immunosuppression of the TME is a primary reason for ICI failure. Bacterial therapy counteracts this by fundamentally reversing the “cold” state, creating an inflamed, “hot” TME receptive to ICIs [80,105]. The mechanisms underpinning this conversion—potent innate immune activation via PAMPs, enhanced antigen presentation, and the reprogramming or depletion of immunosuppressive cells like MDSCs and Tregs—are detailed in “Mechanisms of tumor regulation by engineered bacteria” section. The critical functional outcome in the context of combination therapy is that bacterial intervention creates a permissive microenvironment where preexisting and newly primed T cells can be effectively unleashed by checkpoint blockade.

#### Enhancing antitumor immune response

**Fig. 5.**
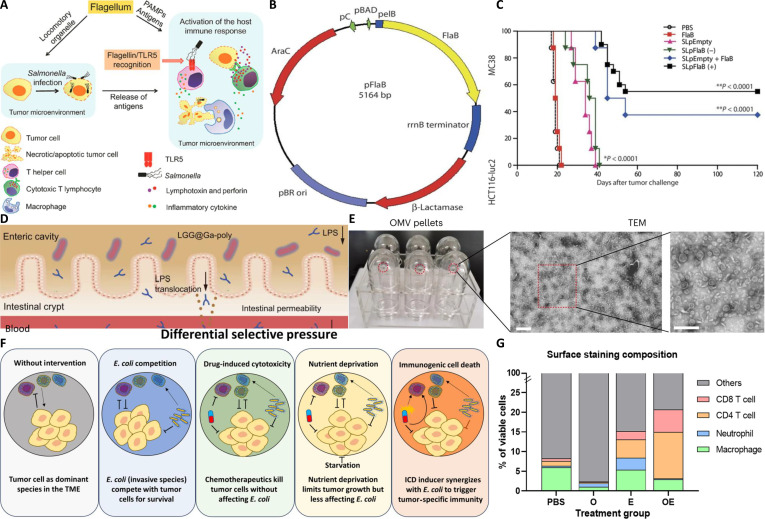
(A) Roles of flagella in *Salmonella*-mediated cancer therapy. (B) Schematic of the engineered plasmid pFlaB (bp, base pair). (C) Kaplan–Meier survival curves of MC38 tumor-bearing mice. **P < 0.0001 versus PBS or SL groups. (D) Transmission electron microscopy (TEM) (scale bar, 200 nm) and cryo-electron microscopy (cryo-EM) (scale bar, 20 nm) images of isolated outer membrane vesicles (OMVs). (E) Cryo-EM image of OMVs. Scale bar, 20 nm. (F) Schematic depiction of tumor immune microenvironment under different treatment scenarios. (G) Comparative analysis of tumor-infiltrating immune cell populations across treatment groups.

Beyond local TME remodeling, bacteria are instrumental in eliciting and sustaining a robust, systemic antitumor immune response, which is crucial for dealing with metastatic disease [78]. First, bacterial colonization and associated cytotoxic activities within tumors promote the release and cross-presentation of tumor antigens, thereby activating robust T-cell responses. Second, engineered bacteria can express and deliver immunostimulatory factors directly into tumors. These factors include cytokines (e.g., IFN-γ, granulocyte-macrophage colony-stimulating factor, and IL-15) and chemokines, which recruit diverse immune cells—including T cells, natural killer cells, and granulocytes—into tumor tissues. For example, *Clostridium* infection recruits granulocytes and cytotoxic lymphocytes into the TME, markedly elevating local cytokine and chemokine levels and thereby promoting tumor eradication [14,106]. Furthermore, bacterial flagellin—a key component of flagella—serves as a potent immunostimulant [107]. Using an attenuated *Salmonella* strain engineered to secrete *Vibrio cholera* flagellin B (FlaB) within tumor tissues [108], researchers effectively suppressed tumor growth and metastasis in murine models. The study demonstrated that FlaB-mediated tumor suppression closely correlates with TLR5-driven host responses in the TME [109]. *Salmonella*-secreted FlaB signals through TLR4 and TLR5, functioning as both a tumor suppressor and an enhancer of antitumor immunity. Moreover, FlaB promotes extensive immune cell infiltration into the TME via TLR4 signaling, collectively augmenting bacterial immunotherapy against cancer [109]. Figure 5A schematically depicts the roles of flagella in *Salmonella*-mediated cancer therapy, emphasizing their dual functions as motile organelles and PAMPs that activate host immune responses via the flagellin/TLR5/nuclear factor κB pathway. Figure 5B presents a schematic of the engineered plasmid pFlaB designed for FlaB expression. Figure 5C demonstrates the efficacy of this approach, presenting Kaplan–Meier survival curves of MC38 tumor-bearing mice. The FlaB-secreting *Salmonella* group (SLpFlaB) showed significantly prolonged survival compared to control groups, indicating potent antitumor effects. Exogenous activation of TLR5 signaling—via recombinant flagellin or its expression—can further improve the efficacy of flagellin-deficient *Salmonella* in melanoma treatment.

#### Combination with ICIs

Despite their revolutionary impact, ICIs targeting pathways such as PD-1/PD-L1 and cytotoxic T-lymphocyte-associated protein 4 are hampered by primary or acquired resistance in a substantial number of patients [110]. Bacterial-based therapies present a promising strategy to circumvent these limitations. The mechanisms through which bacteria counteract ICI resistance are multifaceted [111]: (a) Enhanced tumor antigenicity: Bacteria induce tumor cell lysis and ICD, thereby exposing concealed tumor antigens and augmenting immune-mediated recognition of cancer cells [85]. (b) Promotion of T-cell infiltration: Through the induction of inflammation and chemokine secretion, bacteria facilitate T-cell recruitment into otherwise immunologically “cold” or “immune-excluded” tumors [112], which are typically refractory to ICI monotherapy. (c) Remodeling the immunosuppressive TME: Bacteria can diminish the prevalence and suppressive activity of MDSCs and Tregs, thereby fostering a more permissive TME for ICI function. Preclinical investigations have robustly demonstrated a synergistic effect between bacterial therapies and ICIs [113]. For example, in the MC38 murine tumor model, coadministration of an anti-PD-L1 monoclonal antibody with *Salmonella* resulted in markedly inhibited tumor growth [114]. This combination therapy potently enhances antitumor immune responses and improves therapeutic efficacy, effectively converting ICI-resistant tumors into sensitive ones. Figure 5F schematically illustrates the differential immune responses under various treatment conditions, including bacterial therapy and its combinations with chemotherapeutic agents. Subsequently, Fig. 5G provides a comparative analysis of tumor-infiltrating immune cell profiles across these therapeutic modalities. Clinical trials evaluating such combination regimens are currently in progress, intending to translate these compelling preclinical results into tangible clinical benefits for patients.

#### Modulating the gut microbiota: A systemic synergist for bacterial and immunotherapy

The previous sections have elaborated on the mechanism by which engineered bacteria reshape the TME through localized intratumoral delivery. However, a complete antitumor immune response not only depends on local effects but is also critically regulated by systemic immune status. Among these, the gut microbiota, as the largest immune-regulatory organ in the human body, profoundly influences the baseline level of host systemic antitumor immunity and the response efficiency to ICIs. Therefore, this section will explore how “regulating the gut microbiota” can serve as a systemic and complementary strategy that synergizes with localized intratumoral engineered bacterial therapy and ICIs to collectively overcome immunotherapy resistance. For example, specific probiotics (such as *Bifidobacteria*) have been shown to enhance the efficacy of ICIs, providing a new dimension for the design of combination therapies. While bacterial cancer therapy has traditionally focused on the direct intratumoral delivery of oncolytic bacteria, the gut microbiota’s profound influence on systemic antitumor immunity and immunotherapy efficacy warrants specific discussion [115]. The composition and metabolic output of the gut microbiome significantly modulate a patient’s response to ICIs, establishing it as a crucial therapeutic target [116]. Specific gut bacterial taxa, including *Bifidobacterium*, *Akkermansia muciniphila*, and *Faecalibacterium prausnitzii*, are consistently associated with improved efficacy of PD-1/PD-L1 inhibitors in patients with melanoma and lung cancer. The underlying mechanisms involve immune education, where gut microbes prime systemic immunity by stimulating PRRs on gut-associated immune cells [117], thereby promoting the activation and trafficking of effector T cells to tumors. Additionally, gut bacteria produce immunomodulatory metabolites, such as short-chain fatty acids (e.g., butyrate), which influence immune cell function, cytokine production, and T-cell differentiation [118]. Furthermore, a balanced gut microbiota helps modulate local and systemic inflammation by maintaining intestinal barrier integrity, thereby preventing the translocation of pathogenic bacteria or their products that can provoke detrimental immune responses.

Clinical evidence strongly supports this connection. For example, the oral probiotic formulation CBM588, which contains a live *Bifidobacterium* strain, significantly improved gut microbiota composition in patients with metastatic renal cell carcinoma [119]. When administered alongside ICI therapy, this probiotic intervention markedly extended progression-free survival and resulted in higher disease control rates compared to ICI therapy alone. This finding underscores the direct clinical potential of modulating the gut microbiome to enhance immunotherapy outcomes. In a related context, Fig. 5D and E present transmission electron microscopy and cryo-electron microscopy images of bacterial OMVs, illustrating the potential of using bacterial components—whether derived from commensal gut bacteria or engineered probiotics—as therapeutic agents to modulate host immunity.

The interplay between directly administered tumor-targeting bacteria and the resident gut microbiota is an emerging area of research. Although intratumoral bacterial therapy primarily exerts localized effects, systemic administration may transiently influence gut microbial composition. Conversely, a favorable gut microbiome, fostered through probiotic supplementation or fecal microbiota transplantation, could potentiate the systemic immune responses required for effective bacterial therapy, suggesting a potential synergy. Thus, a dual approach—directly targeting the tumor with engineered bacteria while concurrently optimizing the gut microbiome—holds considerable translational promise for improving outcomes in patients with immunotherapy-resistant cancers.

In summary, the regulation of the gut microbiota and local treatment with engineered bacteria form a “system-local” binary framework for antitumor immune intervention. Future combination therapy strategies may involve a “3-pronged approach”: local intratumoral injection of engineered bacteria to activate in situ immune responses, systemic use of ICIs to relieve T-cell inhibition, and concurrent oral administration of specific probiotics to optimize the overall immune tone and improve intestinal barrier function. This multilevel, multitargeted integration is expected to maximize the translational efficacy of immunotherapy for “cold tumors”.

### Bacteria in combination with targeted therapy, PTT, and photodynamic therapy

**Fig. 6.**
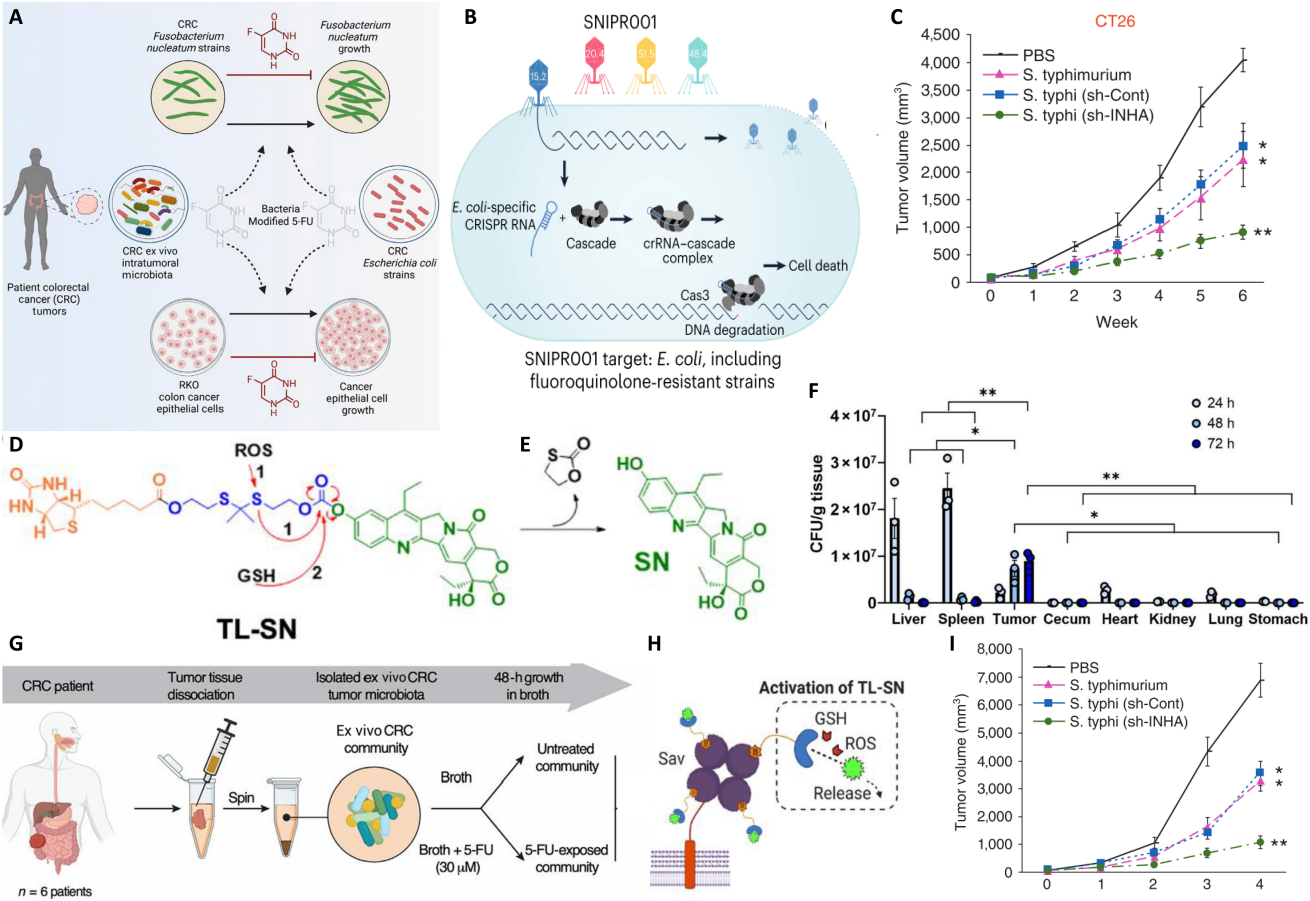
(A) Molecular interaction between OppA protein and heparan sulfate. (B) Composition of SNIPR001, a CRISPR-engineered phage cocktail targeting *Escherichia coli* (*E. coli*). (C) Tumor growth kinetics in CT26 colon cancer models. (D) Establishment of a heterogeneous tumor model using antigen-positive and antigen-negative NXS2 cells. (E) Antitumor efficacy and survival benefit of CAR(NAP) T-cell therapy in vivo. (F) Biodistribution of Lp-CB bacteria in major organs over time (*n* = 3). (G) Workflow for processing patient tumor tissues for microbiota and drug response analysis. (H) Mechanism of action of CAR(NAP) T-cell therapy. (I) Tumor growth kinetics in B16F10 melanoma models. PBS, phosphate-buffered saline; GSH, reduced glutathione.

Bacteria are emerging as a promising platform in oncology, leveraging their unique capacity to selectively target and proliferate inside tumor tissues [120]. This tropism is driven by the hypoxic, acidic, and nutrient-rich TME. Such intrinsic tumor-homing ability makes bacteria ideal vehicles for delivering diverse therapeutic payloads. When combined with targeted therapies, PTT, and photodynamic therapy (PDT), bacterial systems enable more precise and efficient tumor eradication [121]. Bacteria reach tumors via passive or active escape from the bloodstream, followed by local proliferation to achieve targeted accumulation [122]. The figures illustrate various aspects of bacterial applications. Figure 6A illustrates the binding of OppA protein to heparan sulfate, demonstrating how bacteria achieve precise targeting via specific molecular interactions. Figure 6B shows that SNIPR001, composed of 4 complementary CRISPR-aimed bacteriophages, functions as a novel precision antibiotic that specifically targets *E. coli* to prevent bacteremia in hematological cancer patients susceptible to neutropenia. Figure 6C and I show representative data from animal models, such as CT26 colon cancer and B16F10 melanoma, illustrating the pronounced suppression of tumor growth over time, confirming the potent antitumor efficacy of combinatorial therapies. Figure 6D presents a heterogeneous tumor model. Figure 6H delineates the mechanism of CAR(NAP) T-cell therapy. Figure 6E corroborates that bacterial-derived immune stimulants can lead to marked tumor regression and prolonged survival in mice, even in tumors with heterogeneous antigen expression. As illustrated in Fig. 6F, specific bacterial strains show marked enrichment in tumor tissue compared to other mouse organs, highlighting their potential for localized and precise therapeutic interventions. Figure 6G illustrates the workflow for processing of patient tumor tissues for isolation of resident bacterial communities, followed by ex vivo exposure to 5-FU for 48 h and subsequent metagenomic sequencing. The synergy between bacteria and external therapeutic modalities is part of a broader paradigm of developing multifunctional, stimuli-responsive theranostic platforms. In this context, advances in synthetic nanomedicine​ have established a robust framework, particularly with mesoporous silica nanoparticles (MSNs). MSNs are prized for their high surface area, tunable pore size, and ease of functionalization, making them ideal carriers for drug delivery, imaging agents, and photothermal converters [123,124]. The integration of fluorescent carbon dots (C-Dots)​ with MSNs has further yielded “smart” nanocomposites​ capable of integrating real-time optical imaging, photothermal properties, and controlled drug release within a single entity [125,126]. These abiotic platforms exemplify the pursuit of precision and controllability. Inspired by this, engineered bacteria are being explored not merely as alternatives, but as dynamic, living components​ that can actively target tumors, penetrate deeply, and then locally produce or assemble such functional nanomaterials, or directly respond to the same external stimuli (e.g., NIR light) in a synergistic manner [127–129]. The following sections detail how bacteria are harnessed in combination with targeted therapy, PTT, and PDT, within this convergent technological landscape.

#### Bacteria with targeted therapy

Bacteria-based targeted therapy represents a promising strategy for precise tumor eradication [120,122]. This approach involves engineering bacteria to deliver therapeutic agents that specifically target tumor-associated molecules or pathways. By harnessing the living delivery capacity of bacteria, this method addresses key limitations of conventional targeted drugs, including poor tumor penetration and inadequate local drug concentrations. A notable application is the use of bacteria for “suicide gene” therapy. Engineered *Salmonella typhimurium* expressing herpes simplex virus thymidine kinase has been shown to significantly inhibit tumor growth and prolong survival in experimental animal models, while preserving the inherent tumor-targeting and self-amplifying properties of the bacteria [130]. Within the tumor, herpes simplex virus thymidine kinase converts the nontoxic prodrug ganciclovir into a cytotoxic metabolite, enabling localized killing of tumor cells and thereby improving both the specificity and efficacy of the treatment [17]. Bacteria can also function as live carriers for vaccine antigens. For instance, oral or nasal administration of *Salmonella*-based DNA vaccines incorporating the AIDA-1 autotransporter protein has been shown to induce tumor-specific CD4+ and CD8+ cell responses [131]. This significantly enhances the antitumor efficacy of the vaccine, leading to effective suppression of tumor metastasis and growth. Such strategies merge the targeting ability of bacteria with the specificity of immunotherapy, opening new pathways for cancer vaccine development. In the context of TME modulation, engineered bacteria can be designed to deliver enzymes that degrade specific extracellular matrix components. For example, *Salmonella typhimurium* expressing bacterial collagenase (SOT) hydrolyzes type I and IV collagen in a pancreatic ductal adenocarcinoma model [132]. This approach not only suppresses tumor growth but also selectively degrades tumor-associated proteins, thereby enhancing drug penetration and facilitating subsequent therapeutic interventions.

Bacteria are further utilized to deliver gene-silencing agents. *Salmonella* typhi delivering small hairpin RNA against inhibin A exemplifies a promising tumor-targeting strategy [133]. Although inhibin A inhibition alone may only modestly impair cancer cell viability, its combination with bacterial tumor-homing capability holds potential for improving patient survival. Similarly, integration of the *E. coli* tryptophanase operon tnaCAB into the strain *VNP20009* (*VNP20009*-tnaCAB) demonstrated that tryptophanase consumes tryptophan, thereby limiting the production of immunosuppressive kynurenine by IFN-γ-stimulated MDA-MB-468 cancer cells—even in the absence of bacterial replication. This finding underscores the potential of bacteria to disrupt tumor metabolism and reverse immunosuppression, highlighting their utility in metabolic targeting and immune modulation.

Beyond live bacteria, bacterial components such as bacterial ghosts—nonviable bacterial cell envelopes that retain native targeting properties—have emerged as effective delivery vehicles. For example, Mi et al. [134] developed and evaluated DOX-loaded *Salmonella* ghosts, which may provide a novel targeted treatment option for hepatocellular carcinoma patients. More innovative approaches include biohybrid nanoparticles derived from *Bifidobacterium infantis* (Bif@PDA-PTX-NPs), which offer a new direction in tumor therapy [135]. This system utilizes the self-propelled and hypoxia-homing characteristics of *Bifidobacterium* to deliver polydopamine (PDA)-coated paclitaxel nanoparticles (PTX-NPs) precisely to hypoxic or anoxic tumor regions. Bif@PDA-PTX-NPs not only preserve the cytotoxic effect of PTX but also capitalize on the bacterial tropism for hypoxic niches, substantially improving chemotherapeutic outcomes in lung cancer.

#### Bacteria with PTT/PDT

The integration of bacteria with PTT or PDT presents a novel strategy for precise, localized physical tumor ablation. In this synergistic paradigm, engineered bacteria serve as living carriers for photothermal agents or photosensitizers, enabling targeted physical destruction of tumor tissue upon NIR light irradiation [136,137]. Chen et al. [90] investigate a system combining *Salmonella typhimurium VNP20009* coated with PDA for PTT. PDA, a biocompatible photothermal conversion material, is delivered by the bacteria and accumulates specifically within tumors. Upon NIR laser exposure, the enriched PDA generates localized hyperthermia, causing irreversible damage and cell death. This approach capitalizes on the innate tumor-targeting capability of bacteria to achieve high intratumoral concentrations of the photothermal agent, thereby significantly enhancing PTT efficacy while sparing healthy tissues. Experimental results indicated that a single administration of the bacterial construct followed by laser irradiation effectively ablated tumors, with no observed recurrence or metastasis. This methodology demonstrated not only potent tumor eradication but also high precision and minimal invasiveness, highlighting its considerable potential for clinical translation.

The utilization of bacteria as carriers for photothermal agents or photosensitizers offers multiple advantages. Their inherent tumor tropism leads to significant accumulation within the TME, allowing for effective local therapy with reduced systemic dosage [137]. Furthermore, as living organisms, bacteria can proliferate at the tumor site, thereby amplifying the therapeutic payload. Additionally, PTT and PDT are localized treatments with inherently low systemic toxicity. When combined with the targeted delivery afforded by bacteria, this synergy results in a highly favorable therapeutic window. This combination strategy is anticipated to address key limitations of conventional PTT and PDT, such as limited penetration depth and inadequate targeting, providing a promising treatment modality for various solid tumors, including those that are deep-seated.

## Clinical Translation and Challenges of Bacterial Therapy

### Preclinical and clinical research progress

The evolution of bacteria from historical anecdotal observations of tumor regression following infection into a sophisticated engineered therapeutic modality highlights the transformative promise of bacterial-assisted therapy. Numerous bacterial species, each possessing distinct tumor-targeting mechanisms and therapeutic strategies, are currently under investigation in both preclinical and clinical studies. These efforts seek to exploit the capacity of bacteria to selectively colonize tumors, remodel the TME, and deliver therapeutic agents—either as monotherapies or in combination with conventional anticancer treatments. Table S1 summarizes representative bacterial strains in various stages of research and development, detailing their therapeutic strategies, target cancers, key attributes, route of administration, dosage, and current clinical status.

A leading example of bacterial cancer therapy is *Mycobacterium bovis* BCG, which has served as a standard treatment for non-muscle-invasive bladder cancer for decades. Its efficacy stems from the induction of a potent local immune response in the bladder, leading to the eradication of early-stage tumors. The extensive and sustained clinical application of BCG provides strong clinical validation for bacterial immunotherapy. More recently, *Clostridium novyi*-NT, an obligate anaerobe, has been evaluated in clinical trials for its ability to selectively replicate within hypoxic and necrotic regions of solid tumors. This localized replication promotes tumor lysis and elicits inflammatory and immune responses. Its specificity for necrotic tissue restricts growth in oxygenated healthy tissues, mitigating safety risks. *Salmonella typhimurium VNP20009* has been genetically attenuated to reduce toxicity while preserving tumor-homing capability. Early trials of *VNP20009* monotherapy revealed limited efficacy and dose-related toxicities. Current research aims to enhance its safety and therapeutic performance, especially in combination regimens. For example, engineered *Salmonella* strains are increasingly utilized as versatile vehicles for immunomodulators or chemotherapeutic agents, facilitating immune activation or localized drug delivery.

*Listeria monocytogenes* strains such as CRS-207 and ADXS-HER2 represent another engineered platform. For instance, CRS-207 (engineered to express mesothelin) was evaluated in combination regimens for pancreatic cancer. While initial studies showed promising immune responses, subsequent phase II trials did not meet primary survival end points, indicating the challenge of achieving robust efficacy in advanced, immunosuppressive cancers. Ongoing research focuses on optimizing antigen selection and combination partners. Engineered *Bifidobacterium breve* exemplifies a distinct strategy that capitalizes on the anaerobic properties of this commensal bacterium. Orally administered, these strains proliferate specifically within the hypoxic TME, enabling localized delivery of therapeutic proteins or small molecules. The oral administration route offers a noninvasive alternative, improving patient convenience and adherence.

Successful bacterial adjuvant therapies generally share several features: well-defined tumor tropism enabling selective colonization and proliferation within tumors while sparing normal tissues—either inherently, as with anaerobes such as *Clostridium* and facultative anaerobes like *Salmonella*, or via genetic engineering; an engineered safety profile achieved through virulence gene attenuation, auxotrophic modifications (e.g., dependence on nutrients abundant in the TME), and incorporation of suicide switches; and a potent therapeutic mechanism, such as direct oncolysis, immune activation, or targeted drug delivery, capable of altering disease progression. Many advanced bacterial therapies are also designed with synergistic potential, intended for use in combination regimens to address the multifaceted nature of cancer. Conversely, challenges such as those encountered in early *VNP20009* trials often involve insufficient efficacy—where clinical outcomes fall short of preclinical predictions—or dose-limiting toxicity, wherein achieving therapeutic doses without provoking severe systemic adverse events remains difficult. Additionally, the production of live, genetically modified organisms to pharmaceutical standards, coupled with navigating rigorous regulatory pathways, introduces substantial manufacturing and compliance hurdles.

The clinical translation of bacterial therapies offers critical insights that shape future development. The experience with systemically administered strains like *VNP20009* underscores a fundamental challenge: The potent immunostimulation that underlies their efficacy can also lead to dose-limiting toxicities (e.g., cytokine release syndrome) when delivered intravenously, thereby constraining the achievable therapeutic window. This has driven a strategic pivot toward localized delivery routes​ (intratumoral and intravesical) and the engineering of next-generation strains with further-attenuated virulence and enhanced tumor specificity​ to improve safety. Furthermore, the modest efficacy observed in some monotherapy trials highlights that bacteria alone may be insufficient for robust antitumor responses. The future trajectory lies in rationally designed combination regimens, where engineered bacteria act as in situ immune primers, drug factories, or microenvironment modulators to synergize powerfully with established modalities like ICIs, RT, or chemotherapy. For the field to advance, future clinical protocols must rigorously detail and standardize the reporting of administration routes, dosing schedules, and comprehensive safety profiles​ to build a robust, comparative knowledge base that accelerates translational success.

### Safety and toxicity management

The use of live bacteria as therapeutics requires meticulous management of safety and toxicity, stemming from their capacity for self-replication and complex interactions with the host immune system and microbiome. The primary challenges and corresponding mitigation strategies encompass the following key areas:

Balancing the host immune response is a central challenge. PAMPs of therapeutic bacteria, such as lipopolysaccharide and flagellin, can potently activate TLR signaling pathways. While this can induce beneficial antitumor immunity, it may also lead to excessive systemic inflammatory responses, manifesting as cytokine release syndrome with clinical features including high fever, chills, and hypotension. This has been observed in early clinical trials; for instance, the *Salmonella typhimurium* strain *VNP20009* exhibited dose-limiting inflammatory toxicity upon high-dose intravenous administration. To manage this risk, a multifaceted strategy is employed: optimizing the administration route, such as prioritizing intratumoral injection to limit systemic exposure; genetically engineering the bacteria to reduce excessive innate immune activation by deleting highly immunogenic components (e.g., flagellin genes); and implementing clinical supportive care, drawing from experiences with CAR-T cell therapy, where prophylactic or therapeutic use of corticosteroids or IL-6 receptor antagonists (e.g., tocilizumab) can control cytokine release syndrome [138]. Conversely, an overly rapid adaptive immune response may lead to premature bacterial clearance, undermining efficacy. Strategies to address this include using synthetic biology to design genetic circuits that enable bacteria to temporarily “hide” within or modulate the local tumor immune microenvironment.

Ensuring tumor-specific targeting and implementing biocontainment are fundamental to safety. Preventing off-target colonization of bacteria in healthy tissues like the liver or spleen is critical. Strategies to enhance targeting specificity include: (a) auxotrophic engineering, for example, by knocking out genes like aroAor purIto make bacterial proliferation dependent on nutrients abundant in the necrotic tumor core; (b) environment-responsive genetic switches that control bacterial proliferation or therapeutic gene expression using promoters activated by TME cues like hypoxia or low pH; and (c) surface modification to display adhesins targeting tumor-associated antigens. Concurrently, robust biocontainment mechanisms must be integrated. This includes incorporating inducible “suicide switches”, such as lysis genes activated by an exogenous molecule (e.g., arabinose) or by a commonly available antibiotic administered posttreatment, allowing for rapid and complete clearance of the bacteria—a crucial safety feature for any live therapeutic.

Long-term genetic stability, biosafety, and ethical considerations are equally paramount. Risks include reversion to virulence through mutations within the host and horizontal gene transfer of engineered constructs to commensal bacteria. Mitigation measures involve using stable chromosomal integration rather than plasmid-based expression systems, employing antibiotic-free selection markers, and performing large deletions of virulence genes instead of point mutations. From a regulatory perspective, these therapies are classified as Live Biotherapeutic Products and must adhere to specific guidelines from agencies like the Food and Drug Administration and the European Medicines Agency. This necessitates stringent biocontainment protocols throughout the entire chain of manufacturing, storage, transportation, and administration. Ethical review of clinical trials must emphasize comprehensive informed consent regarding the risks of novel therapies, equitable patient selection, and the establishment of long-term follow-up plans to monitor delayed effects. Transparent scientific communication is also vital for building public trust.

### Manufacturing and quality control

The production and quality control of live bacterial therapeutics pose unique challenges compared to conventional pharmaceuticals and other biologics. Ensuring the safety, purity, potency, and consistent quality of a living, replicating product requires specialized infrastructure, analytical methods, and regulatory frameworks.

Manufacturing faces several inherent obstacles, including achieving batch-to-batch consistency in viability, cell count, genetic stability, and therapeutic transgene expression—a particular challenge given the natural variability of living systems. Maintaining sterility is paramount; although the product itself is a microorganism, it must be rigorously free from contaminating species, necessitating aseptic processing and stringent environmental controls. Stability and shelf-life determination present further difficulties, as live bacteria typically require cryopreservation or lyophilization to maintain viability and function over time. Scaling production from laboratory to commercial scale demands specialized bioreactors and downstream processes that preserve bacterial integrity. Finally, ensuring genetic stability—preventing mutation or functional loss during production and storage—remains an ongoing concern. Regulatory agencies such as the Food and Drug Administration and European Medicines Agency have begun developing specific guidelines for Live Biotherapeutic Products to address their unique characteristics. Key quality control parameters and regulatory expectations include rigorous identity and purity testing through methods such as 16S ribosomal RNA gene sequencing or whole-genome sequencing for strain confirmation, coupled with stringent screening for adventitious agents. Accurate enumeration of viable cells, typically via colony-forming unit assays, along with functional assays that confirm therapeutic activity—such as expression of therapeutic proteins, oncolytic capacity, or immune activation potential—are essential for assessing viability and potency. Comprehensive genetic characterization through genomic sequencing is required to verify the presence and stability of engineered modifications, confirm the absence of unintended mutations, and fully characterize antibiotic resistance profiles. Furthermore, developing robust pharmacokinetic and pharmacodynamic assays is crucial for monitoring in vivo bacterial distribution, persistence, and activity, thereby informing optimal dosing regimens. Establishing clear and justified release specifications for each product batch is critical to ensure consistency and patient safety. Adherence to stringent Good Manufacturing Practice guidelines, tailored specifically for biological products, is mandated across all aspects of facilities, equipment, personnel, and documentation. Lastly, comprehensive postmarket surveillance is necessary to continuously monitor product quality, safety, and efficacy following clinical approval, including long-term follow-up of treated patients. Addressing these intricate manufacturing and quality control challenges is essential to establishing bacterial therapy as a safe, effective, and reliable treatment modality, thereby facilitating its broader integration into oncology practice.

Looking forward, the convergence of synthetic biology with artificial intelligence (AI) and machine learning is poised to revolutionize the design and deployment of bacterial therapeutics. Concrete use cases include: (a) using AI algorithms to predict optimal bacterial chassis and gene circuit designs for specific tumor types; (b) applying machine learning models to analyze complex microbiome data, thereby identifying patient-specific biomarkers for therapy response and guiding personalized combination regimens; and (c) leveraging computational tools to simulate bacterial population dynamics within the TME, which can inform dosing schedules and predict therapeutic outcomes [139,140]. These approaches will significantly accelerate the iterative design cycle, from in silico modeling to in vivo application.

## Conclusions and Future Perspectives

This review explores bacteria-mediated cancer therapy, highlighting its diverse antitumor mechanisms and synergy with conventional treatments. Key mechanisms include inherent tumor tropism, direct oncolysis, and sophisticated modulation of the TME, which enhance therapeutic payload delivery and help overcome treatment resistance. Despite challenges in safety, manufacturing, and regulation, the field is advancing toward transformative clinical applications.

A critical future direction involves refining the safety and efficacy of bacterial therapies. This will leverage advanced genetic engineering to achieve precise tumor targeting, controlled bacterial proliferation, and dynamic therapeutic delivery, thereby minimizing off-target effects and maximizing antitumor efficacy. A key strategy is to balance the host immune response to prevent both excessive inflammation and premature bacterial clearance. Another priority is to address the manufacturing and regulatory complexities specific to live biotherapeutic products. It is essential to develop scalable, consistent, and cost-effective manufacturing processes, supported by robust quality control and tailored regulatory frameworks. Furthermore, the integration of AI and machine learning holds substantial promise for optimizing bacterial strain design, predicting in vivo behavior, and ensuring product consistency, as discussed in the context of future engineering and manufacturing paradigms.

The full potential of bacterial therapy will be realized through its integration into personalized cancer treatment paradigms. This includes patient selection based on tumor microbiome profiling and the rational design of combinations with immunotherapies. Furthermore, nonliving bacterial derivatives, such as OMVs, represent promising alternatives with improved safety profiles and easier regulatory paths, facilitating their clinical translation.

## Data Availability

The data that support the findings of this study are available from the corresponding authors upon reasonable request.

## Supplementary Materials

Supplementary 1 Table S1 References [141–151]
