# Supplementary material for: Biomaterials Based on Bacteria for Cancer Clinical Therapy
Source: Biomater Res. 2026 Apr 13;30:0350. doi: 10.34133/bmr.0350 (PMC13074759; doi:10.34133/bmr.0350)
Supplement: Supplementary 1 — Table S1 References [141–151] [file bmr.0350.f1.docx]

**Table S1**

Research and development stages of bacterial adjuvant therapy and an overview of representative projects in cancer treatment

| Development Phase | Representative Strain/Therapy | Administration Route & Regimen | Therapeutic Strategy | Targeted Cancer Type | | Key Features | Current Status | | Reference |
| --- | --- | --- | --- | --- | --- | --- | --- | --- | --- |
| Marketed/Clinical Use | *Mycobacterium bovis* BCG | Route: Intravesical instillation.  Regimen: Induction therapy (typically weekly for 6 weeks) followed by maintenance schedules. | immune stimulation | Bladder Cancer | The first and most successful bacterial immunotherapy, used as standard treatment for non-muscle-invasive bladder cancer. | | | Standard Therapy | [141] |
| Clinical Trial Phase | *Clostridium novyi-NT* | Route: Direct intratumoral injection.  Regimen: Single or repeated injections into clinically accessible tumor lesions. | oncolytic/anaerobic-specific colonization | Solid tumors, such as sarcomas and melanomas. | Dissolving the tumor necrotic area causes intense local inflammation and an immune response. | | | Phase II | [142] |
|  | *Salmonella typhimurium* *VNP20009* | Route: Intravenous infusion.  Regimen: Evaluated in dose-escalation studies (multiple infusions over a treatment cycle). | immunostimulant/chemotherapy drug delivery | metastatic melanoma, renal cell carcinoma | Reduced toxicity, tumor-targeting, and designed to activate the immune system or deliver drugs. | | | Phase II | [143] |
|  | *Listeria monocytogenes* (CRS-207, ADXS-HER2) | Route: Intravenous.  Regimen: Administered in prime-boost schedules, often in combination with chemotherapy (e.g., gemcitabine) or other vaccines. | immune activation/antigen presentation | pancreatic cancer, malignant pleural mesothelioma | After modification, it is used to present tumor antigens and stimulate T-cell immune response. | | | Phase II | [144] |
|  | *Bifidobacterium breve(engineering version)* | Route: Oral administration or intratumoral injection (preclinical).  Regimen: Explored as repeated oral gavage or direct injection in murine models. | immunomodulation/drug delivery | solid tumor | Oral administration, capable of specifically proliferating in the hypoxic microenvironment of tumors, can be engineered to express cytokines (such as IL-2) or used for local delivery of chemotherapeutic drugs (such as Bif@DOX-NPs) to enhance efficacy and reduce toxicity. | | | Phase II | [145] |
|  | *E. coli Nissle 1917(engineering version)* | Route: Intratumoral injection.  Regimen: Repeated injections (e.g., administered weekly). | prodrug conversion/gene delivery | Hepatocellular carcinoma, colorectal cancer | By engineering enzymes, non-toxic prodrugs are converted into toxic drugs to achieve localized killing. | | | Phase I | [146] |
|  | oncolytic streptococcus | Route: Intratumoral injection.  Regimen: Explored in single or multiple-injection protocols in preclinical models. | immune activation/tumor necrosis | head and neck cancer, pleural effusion, lymphangioma | By inducing local inflammatory responses and activating host immune cells (such as NK cells, macrophages), leading to tumor necrosis. | | | Phase I | [147] |
| Basic Research/Preclinical Phase | *F. nucleatum* (LipoFM-CPG) | Route: Intravenous (as a bacterial component-based vaccine).  Regimen: Investigated as a prophylactic or therapeutic vaccine in immunization schedules. | immune clearance/reversal of resistance | Colorectal cancer and other *F. nucleatum*-related tumors | Induce specific CD8+ T cell responses and humoral immune responses, clear *F. nucleatum* within tumors, thereby reversing the chemotherapy resistance it induces. | | |  | [148] |
|  | *Pseudomonas aeruginosa*, *Bacillus subtilis* | Route: Intratumoral injection.  Regimen: Preclinical studies utilizing engineered toxin delivery systems. | Diverse strategies (oncolysis, immune modulation, gene therapy) | various solid tumor models | Explore the tumor tropism, safety, and genetic modification potential of new strains, and develop more efficient and safer treatment models. | | |  | [149] |
|  | Intelligent response to bacteria (such as through biosensors, feedback loops) |  | precise drug release/multimodal synergy | Various in vitro/in vivo tumor models | Based on synthetic biology, engineered bacteria can sense specific signals in the TME, such as hypoxia and pH levels, and release therapeutic molecules or modulate their functions on demand, achieving highly controllable and precise treatment. | | |  | [150] |
|  | Phage-guided bio-inorganic hybrid nanosystem |  | Microbial reshaping enhances chemotherapy efficacy. | colorectal cancer | Bacteriophage targeting the elimination of pro-tumor *F. nucleatum*; nanomaterials (IDNPs) promote the growth of anti-tumor bacteria, such as butyrate producers. Synergistic chemotherapy. | | |  | [151] |
